# High-concentration boron doping of graphene nanoplatelets by simple thermal annealing and their supercapacitive properties

**DOI:** 10.1038/srep09817

**Published:** 2015-05-05

**Authors:** Da-Young Yeom, Woojin Jeon, Nguyen Dien Kha Tu, So Young Yeo, Sang-Soo Lee, Bong June Sung, Hyejung Chang, Jung Ah Lim, Heesuk Kim

**Affiliations:** 1 Photo-electronic Hybrids Research Center, Korea Institute of Science and Technology (KIST), Seoul 136-791, Korea; 2 Department of Chemistry, Sogang University, Seoul 121-742, Korea; 3 Nano-Materials and Engineering, Korea University of Science and Technology (UST), Dae-Jeon, Korea; 4 Interface Control Research Center, Korea Institute of Science and Technology (KIST), Seoul 136-791, Korea; 5 KU-KIST Graduate School of Converging Science and Technology, Korea University, Seoul 136-701, Korea; 6 Advanced Analysis Center, Korea Institute of Science and Technology (KIST), Seoul 136-791, Korea

## Abstract

For the utilization of graphene in various energy storage and conversion applications, it must be synthesized in bulk with reliable and controllable electrical properties. Although nitrogen-doped graphene shows a high doping efficiency, its electrical properties can be easily affected by oxygen and water impurities from the environment. We here report that boron-doped graphene nanoplatelets with desirable electrical properties can be prepared by the simultaneous reduction and boron-doping of graphene oxide (GO) at a high annealing temperature. B-doped graphene nanoplatelets prepared at 1000 °C show a maximum boron concentration of 6.04 ± 1.44 at %, which is the highest value among B-doped graphenes prepared using various methods. With well-mixed GO and g-B_2_O_3_ as the dopant, highly uniform doping is achieved for potentially gram-scale production. In addition, as a proof-of-concept, highly B-doped graphene nanoplatelets were used as an electrode of an electrochemical double-layer capacitor (EDLC) and showed an excellent specific capacitance value of 448 F/g in an aqueous electrolyte without additional conductive additives. We believe that B-doped graphene nanoplatelets can also be used in other applications such as electrocatalyst and nano-electronics because of their reliable and controllable electrical properties regardless of the outer environment.

Graphene is one of the most promising materials for nanoelectronic applications including transparent conductive electrodes, electrocatalysts, field effect transistors, chemical/biological sensing platforms and energy devices because of its large surface area, high chemical stability and mechanical or electrical robustness. For this reason, the demand for graphene has increased dramatically in recent years^1^,^2^,^3^,^4^. One interesting feature of graphene is that it has a unique electronic structure with zero band-gap, leading to simultaneous semiconducting and semi-metallic properties. To realize graphene-based nanoelectronics, it is of much importance to tune the electrical properties of graphene in accord with the desired application.

One of the most promising routes to modulate the electrical properties of graphene is the chemical substitution of carbon atoms in *sp*^2^-bonded carbon networks with hetero atoms such as boron or nitrogen, which is called substitutional doping^5^,^6^,^7^,^8^,^9^. Previously, graphenes doped with hetero atoms have been produced either by chemical vapor deposition (CVD)^10^,^11^,^12^,^13^, by arc discharge, by metal-organic frameworks (MOFs)/or covalent organic frameworks (COFs) as hetero atoms-doped graphene templates^14^,^15^,^16^,^17^,^18^, or by chemical treatment of reduced graphene oxide (rGO)/or graphene oxide (GO). Among them, CVD is the most common method and has been extensively used to produce doped graphene. During CVD, the carbon atoms are effectively and uniformly replaced by hetero-atoms such as boron or nitrogen with a high doping level (e.g., 4.3 at % boron or 9 at % nitrogen)^6^,^10^,^11^,^12^,^13^. While the doped graphene produced by CVD is certainly applicable for thin film-based electronic devices, it is unsuitable for application as an electrode in energy storage devices requiring three-dimensional nanostructure and it is difficult to incorporate such graphene into facile bulk solution processes.

On the other hand, the chemical treatment of rGO or GO has advantages for bulk synthesis and incorporation into a solution process, which is quite beneficial for the easy formation of structural electrodes for energy applications. However, the doped graphene nanoplatelets produced from rGO or GO have poor doping uniformity and low doping efficiency. Therefore, investigations into techniques for substitutional doping of graphene nanoplatelets suitable for bulk synthesis and uniform doping with high efficiency are necessary to improve the electrical properties of doped graphene nanoplatelets and expand their applications into energy-related materials.

Most studies on substitutional doping of graphene nanoplatelets using GO or rGO as starting materials have focused on nitrogen-doped graphenes. N-doped graphene nanoplatelets have been extensively synthesized by the treatment of GO with nitrogen-containing chemicals such as ammonia and urea, and doping efficiencies of approximately 5–8.05 at % have been achieved^19^,^20^,^21^,^22^. However, the electrical properties of N-doped graphene are very sensitive to the adsorption of oxygen and water molecules from the atmosphere, because these molecules act as electron-withdrawing groups and thus attenuate the effect of nitrogen doping. In fact, some reports have demonstrated the change of N-doped graphene from n-type to p-type characteristics due to the adsorption of water and oxygen molecules^23^,^24^. Therefore, it is important to investigate boron-doped graphene nanoplatelets, which would have reliable electrical properties regardless of the outer environment. There have been only a few reports on B-doped graphene nanoplatelets because of difficulties in their synthesis. Wu *et al.* reported that rGO can be doped with boron from boron trichloride (BCl_3_) gas by heat treatment, but only a low efficiency of 0.88 at % boron doping was obtained^25^. More recently, Han *et al.* reported that B-doped graphene nanoplatelets could be synthesized by the reaction of GO with a borane-tetrahydrofuran adduct (BH_3_-THF) under reflux in aqueous solution. However, the reaction time was too long, and the boron concentration was quite low at 1.1 at %^26^.

In this study, we demonstrated that B-doped graphene nanoplatelets can be prepared by simple thermal annealing of GO nanoplatelets combined with boron oxide (B_2_O_3_). The GO/B_2_O_3_ mixture was annealed at various temperatures to systematically control the efficiency of boron doping and the degree of reduction of GO. B-doped graphene nanoplatelets prepared at 1000 °C showed the maximum boron concentration of 6.04 ± 1.44 at % which is the highest value among B-doped graphenes produced by CVD, arc discharge or the substitutional doping of GO or rGO. In addition, B-doped graphene nanoplatelets show a uniform distribution of boron, indicating that this simple method is very useful for bulk synthesis of B-doped graphene nanoplatelets with uniform high-concentration boron doping. Finally, as a proof-of-concept, we have also demonstrated highly B-doped graphene nanoplatelets as an electrode in an electrochemical double-layer capacitor (EDLC), indicating their potential for use in energy storage applications. Highly B-doped graphene nanoplatelets prepared at 1000 °C showed excellent specific capacitance value of 448 F/g in an aqueous electrolyte, which is 3-folds higher than that of a thermally reduced GO electrode without boron (135 F/g). The improved specific capacitance of B-doped graphene nanoplatelets is due to their great enhancement in electrical conductivity and specific surface.

## Results and Discussion

**Figure 1:**
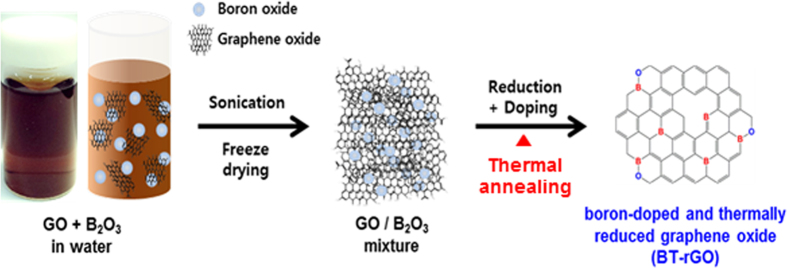
Schematic illustration of the preparation of BT-rGO.

Graphene oxide (GO) obtained by the oxidation and exfoliation of graphite is the most promising candidate as a starting material for bulk synthesis of doped graphene nanoplatelets. Fig. 1 illustrates the preparation of B-doped graphene nanoplatelets (BT-rGO) by simple thermal annealing of a GO/B_2_O_3_ mixture. The BT-rGO was prepared in two steps: the formation of boron oxide aqueous solution well mixed with GO by ultrasonication and the thermal annealing of the GO/B_2_O_3_ mixture after freeze-drying for simultaneous reduction and doping. We found that the GO/B_2_O_3_ aqueous solution was very clear with a dark-brown color, which means that the solution was very homogeneous. This homogeneity leads to the obtained ultra-uniform doping of boron into the network of the graphene nanoplatelet. Thermally reduced graphene oxide without boron oxide (T-rGO) was also prepared as a control.

**Figure 2:**
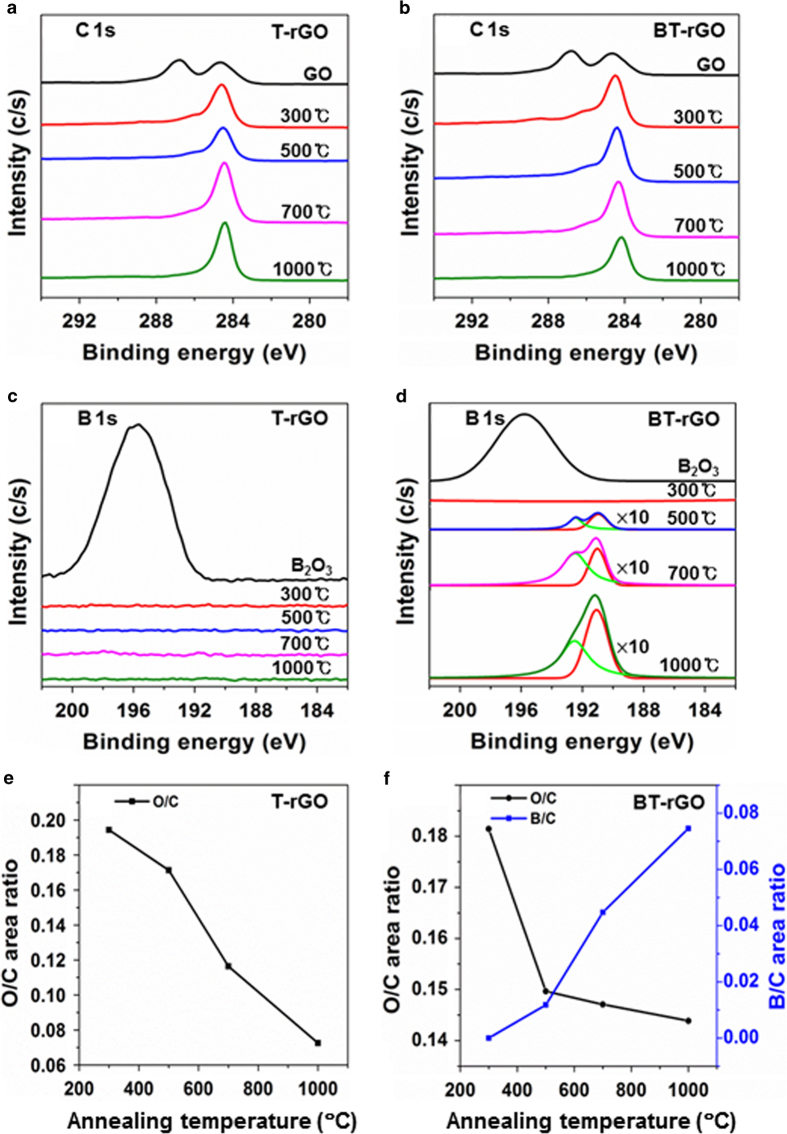
XPS high-resolution spectra of (**a**) C(1s) and (**c**) B(1s) peaks of T-rGO and (**b**) C(1s) and (**d**) B(1s) peaks of BT-rGO. (**e**) O/C area ratio of T-rGO and (**f**) O/C and B/C area ratios of BT-rGO.

BT-rGO and T-rGO samples annealed at various temperatures were quantitatively and qualitatively characterized by X-ray photoelectron spectroscopy measurements (XPS). Figure S1 in the supporting information (SI) shows the XPS survey spectra of T-rGO and BT-rGO. As the annealing temperature increases from 300 to 1000 °C, the oxygen peaks of T-rGO and BT-rGO at ~530 eV decrease owing to the reduction of GO. In addition, a tiny boron peak at ~189 eV due to the boron doping starts to appear. In order to analyze the species of functional groups that form as the annealing temperature increases, high resolution XPS spectra were obtained. Fig. 2(a) shows the C(1s) peaks as a function of annealing temperature of T-rGO and BT-rGO. Before thermal annealing, as-prepared GO is characterized by a C(1s) peak at 284.5 eV, and two distinct peaks at 286.6 and 288.1 eV. The peak at 284.5 eV arises from the non-oxygenated ring C (C-C bonds), whereas the two peaks at 286.6 and 288.1 eV arise from the C in C-O bonds of hydroxyl and 1, 2-epoxide functionalities and from the carbonyl C in C = O bonds of carboxyl and ketone functionalities, respectively^9^,^27^,^28^. After thermal annealing, the intensities of the C(1s) peaks at 286.6 and 288.1 eV are much smaller than those of GO. As the temperature increases, the peak intensities at 286.6 and 288.1 eV decrease, indicating that GO is more reduced at higher temperatures. This is because most of the carboxyl groups in GO decompose at 200–600 °C, and the residual carboxyl and partial hydroxyl groups decompose at ~800 °C^29^. In addition, residual hydroxyl and partial epoxide groups decompose at ~1000 °C 29. The B(1s) peaks of T-rGO and BT-rGO are shown in Fig. 2(c). While T-rGO shows no boron peak, BT-rGO is characterized by a B(1s) peak at 190.8 eV with a strong shoulder near 192.6 eV. This B(1s) peak is different from that of B_2_O_3_ at 196.0 eV, demonstrating that boron atoms are linked with carbon atoms in the *sp*^2^ carbon network of rGO. The peaks at 190.8 and 192.6 eV arise from the B atoms of BC_3_ and BC_2_O, respectively^25^. As the annealing temperature increases from 500 to 1000 °C, the peak intensities at 190.8 and 192.6 eV increase and the peak from BC_3_ at 190.8 eV becomes more predominant at 1000 °C. This demonstrates the formation of more B-C bonds in carbon network and the reduction of more oxygen groups with increasing temperature.

The boron source used in this study is an amorphous glass-like B_2_O_3,_ as confirmed by X-ray diffraction (XRD) measurement (Figure S2(a) in SI)^30^. Basically, two crystalline phases (α-B_2_O_3_ and β-B_2_O_3_) and a g-B_2_O_3_ glass-like phase of boron (III) oxide are known to exist, where the local structure of g-B_2_O_3_ is closest to the structure of α-B_2_O_3_^31^,^32^. Two characteristic wide halos in the XRD data of g-B_2_O_3_ shift toward lower 2Θ positions with increasing temperature. In particular, the interplanar spacings of g-B_2_O_3_ calculated from the first halo increases from 4.00 Å (2Θ = 22.2°) at 25 °C to 4.48 Å (2Θ = 19.8°) at 1000 °C. Furthermore, thermogravimetric analysis (TGA) of g-B_2_O_3_ (Figure S2(b)-(d) in SI) shows a weight loss at more than 500 °C. This could be from partial decomposition of g-B_2_O_3_ rather than boiling because the boiling temperature of g-B_2_O_3_ is ~1500 °C. At 1000 °C, approximately 6% by weight of g-B_2_O_3_ has decomposed. Thus, during our synthesis of doped graphene nanoplatelets, both the oxygen groups of GO are removed and g-B_2_O_3_ is decomposed as the annealing temperature increases up to 1000 °C. The defects derived from the removal of oxygen groups could be active sites for the chemical substitution of carbon atoms by boron atoms. In particular, the boron atoms by the decomposition of g-B_2_O_3_ could easily react with the carbon atoms at these sites.

For quantitative characterization, the O/C area ratios and B/C area ratios of T-rGO and BT-rGO were calculated from the XPS data (Fig. 2(e)-(f)) by measuring the ratio of the peak areas and correcting for the sensitivity factors to obtain a corrected peak area ratio. The corrected O/C area ratios of T-rGO and BT-rGO decrease with increasing temperature, thus implying the reduction of oxygen groups. On the other hand, the corrected B/C area ratio of BT-rGO increases up to 0.075 for the annealing temperature of 1000 °C. This demonstrates that the efficiency of boron doping and the degree of reduction of GO could be systematically controlled by simple thermal annealing. The absolute amount of boron doped at 1000 °C, not the relative boron amount versus carbon amount, was measured as 6.04 ± 1.44 at % by XPS. This is the highest value among B-doped graphenes synthesized by CVD, arc discharge or substitutional doping of GO or rGO.

**Figure 3:**
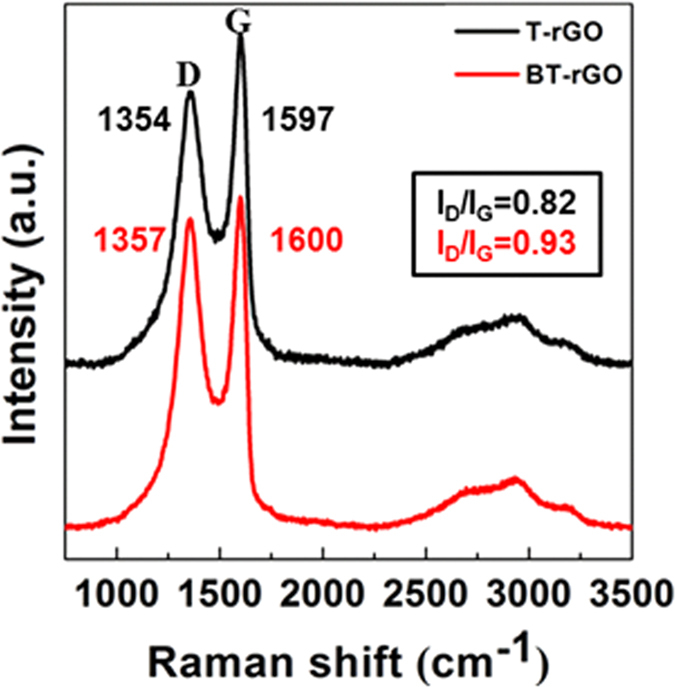
Raman spectra of T-rGO and BT-rGO annealed at 1000 °C.

**Figure 4:**
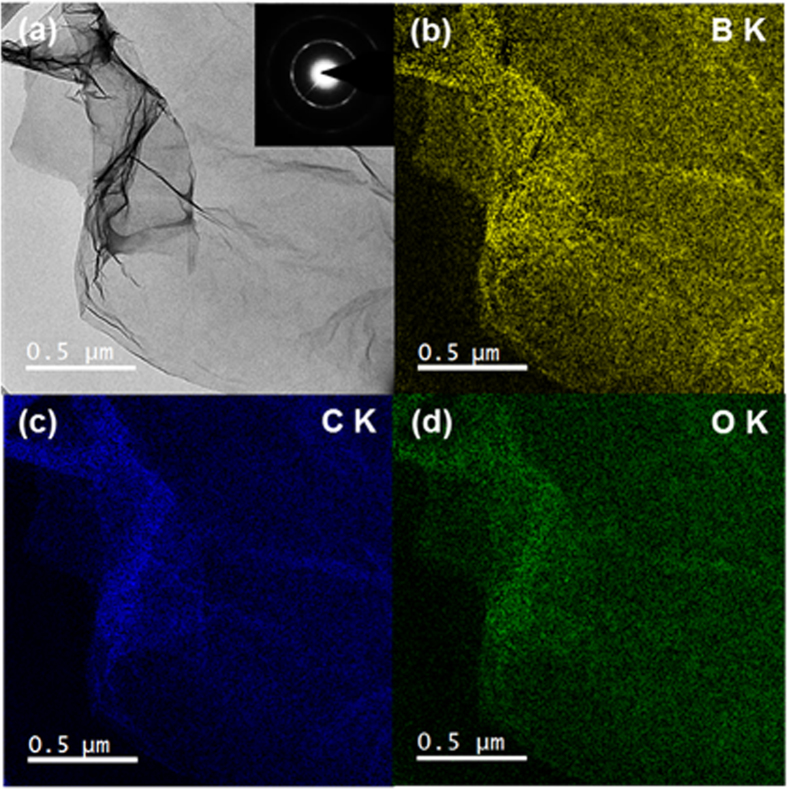
TEM and EELS mapping images of BT-rGO annealed at 1000 °C: (**a**) elastic TEM image and (**b**) boron, (**c**) carbon, and (**d**) oxygen EELS mappings.

Raman spectroscopy is the most powerful and non-destructive technique for identifying the structure and quality of graphene^3^,^9^,^33^,^34^. As shown in Fig. 3, BT-rGO annealed at 1000 °C shows an intense G band at 1600 cm^−1^ and a wide 2D band at 2699 cm^−1^, indicating that BT-rGO has a graphite structure with a few layers. Compared to T-rGO prepared at the same temperature, the G band of BT-rGO is slightly shifted to higher frequency from 1597 to 1600 cm^−1^ and its full width at half-maximum (FWHM) is also slightly increased from 88.7 to 89.0 cm^−1^. This confirms the boron doping as reported in the literature^9^,^33^. BT-rGO also shows a strong D band at 1357 cm^−1^ due to the many defects derived from insufficient reduction and boron doping in the structure, as well as a higher intensity ratio of D and G band (I_D_/I_G_) of 0.93, compared to 0.82 for T-rGO. The in-plane crystallite sizes calculated using the equation reported in the literature^9^ for T-rGO and BT-rGO are 21 and 18 nm, respectively. The smaller crystallite sizes for doped graphene nanoplatelets than for undoped graphene nanoplatelets indicate that the BT-rGO possesses more defects because of the boron doping^9^. As shown by the Raman analysis, BT-rGO has few-layered nanostructures. This is also confirmed by atomic force microscopy (AFM) images (Figure S3 in SI), which show a two-dimensional structure of BT-rGO with an average thickness of 1.5 nm, implying a nanostructure with a few layers. This average thickness is slightly higher than that of GO (0.8 ~ 1.2 nm), which means that the GO sheets restack during thermal reduction. The transmission electron microscopy (TEM) image in Fig. 4(a), also shows the restacked structure of BT-rGO. The interlayer spacing of BT-rGO is approximately 3.7–3.8 Å, as calculated from the 2Θ = 23°–24° peak in the XRD data (Figure S4 in SI). This interlayer spacing is slightly higher than the (002) graphite spacing of 3.36 Å, indicating a structure similar to that of graphite with more defects derived from the boron doping^35^.

The boron atoms on the basal plane of BT-rGO are uniformly distributed, which is confirmed by the electron energy loss spectroscopy (EELS) mapping. Fig. 4(b)–4(d) shows the distribution of boron, carbon and oxygen elements in the plane of BT-rGO annealed at 1000 °C. These data demonstrate the uniform doping of boron atoms into carbon networks of graphene nanoplatelets derived from the homogeneous mixing of GO with B_2_O_3_. The atomic percentage of boron determined from the EELS mapping is 6.88%, which is approximately consistent with that from the XPS analysis.

Finally, we investigated the effect of boron doping on the electrical properties of BT-rGO and demonstrated, as a proof-of-concept, a highly B-doped rGO electrode in an electrochemical double-layer capacitor (EDLC) to confirm the potential of this material for use in energy storage applications. The current-voltage (I-V) curves in Figure S5(a) in the SI show a conspicuous difference between the electrical conductance of BT-rGO annealed at 1000 °C and that of T-rGO prepared at the same temperature. Furthermore, the electrical conductivity of BT-rGO shown in Figure S5(b) is 44.34 S/cm, which is one order of magnitude higher than that of T-rGO (1.81 S/cm). GO is essentially an insulator, so deoxygenation and reduction are imperative to guarantee an electrically conducting nature. As mentioned above, with increasing annealing temperature, the degree of reduction of GO increases, which results in a gradual improvement in conductivity, as shown in Figure S5(b). Notably, the drastically higher conductivity of BT-rGO compared to that of T-rGO at each annealing temperature implies that the boron doping is more dominant than thermal reduction of GO in terms of affecting the electrical conductivity. It is known that doping increases the carrier concentration of materials, thus enhancing the electrical conductivity^9^,^26^.

**Figure 5:**
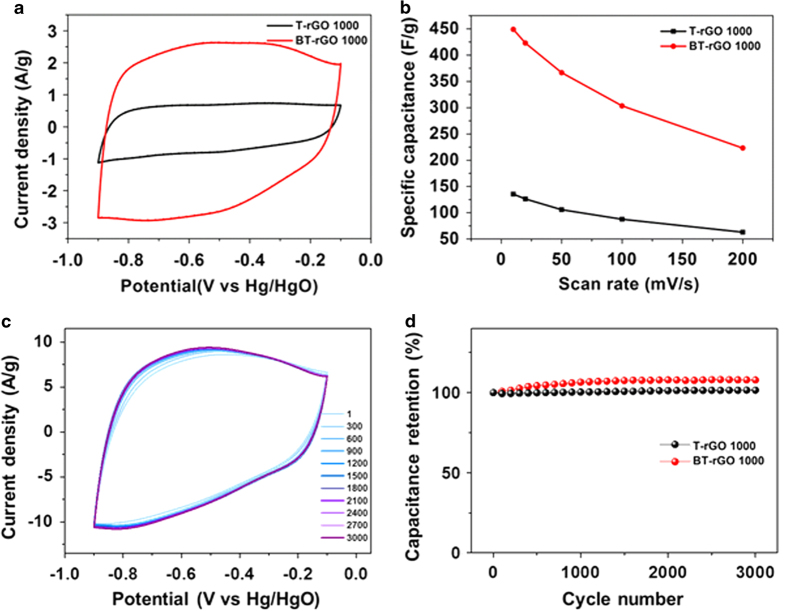
(**a**) CV curves at a scan rate of 10 mV/s, (**b**) specific capacitances as a function of scan rate, (**c**) CV curves for 3000 cycles at a scan rate of 50 mV/s, and (**d**) capacitance retentions as a function of cycle number of T-rGO and BT-rGO electrodes.

Graphene has many inherent advantages as an electrode of a supercapacitor, such as its high surface area, high electrical conductivity, light weight, and excellent flexibility^36^,^37^. Recently, supercapacitors based on N- or B- doped graphene electrode have exhibited higher capacitance than those based on undoped graphene electrodes because of both their enhanced electrical properties and their higher interfacial capacitance^21^,^38^,^39^. Fig. 5(a) shows the cyclic voltammetry (CV) curves of T-rGO and BT-rGO annealed at 1000 °C at a scan rate of 10 mV/s obtained by three-electrode cell measurement using a 6 M KOH electrolyte. The BT-rGO electrode has a much higher current density than the T-rGO electrode, indicating a large increase in capacitance due to the B doping. The near-rectangular CV curve for the T-rGO electrode indicates typical supercapacitor behavior. By contrast, a rectangular shape with a pair of wide humps is observed in CV curve of BT-rGO. This means that the capacitive response in the BT-rGO electrode is a combination of electric-double layer capacitance and reversible faradic reactions involving proton exchange. Although the detailed mechanism of the faradaic reaction in the BT-rGO cell is not fully understood, the B-doping of rGO may be responsible for a redox reaction. It has been reported that the presence of B- and/or O-containing functional groups in carbon materials might result in electrochemical redox reactions on the surface^26^,^40^. As shown in Fig. 5(b), the calculated specific capacitance of the BT-rGO electrode at a scan rate of 10 mV/s based on the total mass of the active electrode materials is 448 F/g, which is 3-fold higher than that of the T-rGO electrode (135 F/g). This is close to the highest capacitance value obtained for any graphene-based supercapacitor (456 F/g) reported by Yan *et al.*, even though no conductive additives such as carbon black were used^41^. The dependence of the specific capacitance values on the scan rate clearly demonstrates that the BT-rGO electrodes exhibit significantly better capacitance performance than undoped T-rGO electrodes. Scan rate-dependency on the specific capacitance was observed for both BT-rGO and T-rGO electrodes. As increasing a scan rate from 10 to 200 mV/s, the specific capacitance of BT-rGO electrode decreases to 50% of the capacitance at a scan rate of 10 mV/s. For T-rGO electrode, more than 55% decrease of the capacitance is also observed. The rate-dependent capacitance may be attributed to rate-dependent ion diffusion behavior in carbon materials. At low rates, ions can penetrate into the inner surfaces of carbon materials, whereas at high rates, only the exterior surface is accessible^40^. Additionally, we speculate that the residual NMP solvent at the nano-sized pores in rGO materials may affect the ion accessibility to the electrode surface in charge/discharge process. In fact, as shown in Figure S6 in the SI, the CV curve of BT-rGO electrode is significantly improved when the electrode is soaked in DI water for 12 h before CV measurement, because the residual NMP solvent is replaced with water^42^. The electrochemical stability of BT-rGO electrode was measured by repeating CV test for 3000 cycles at a scan rate of 50 mV/s. As shown in Fig. 5(c), no distortion of CV curves is observed after the cycle test. Fig. 5(d) exhibits that the capacitance of BT- rGO electrode retains without degradation until 3000 cycles. These results indicate that BT-rGO is a stable electrode material with good cycle-life stability.

The specific capacitance of a supercapacitor is mainly affected by the electrical conductivity and specific surface area of the electrode. As mentioned above, the great enhancement in the electrical conductivity of the BT-rGO electrode definitely contributes to a reduction in the resistive loss in the capacitor circuit, and thus using a doped electrode can improve the capacitance without the use of conductive additives. Furthermore, Brunauer-Emmett-Teller (BET) N_2_ adsorption measurements were performed to investigate the specific surface area of the BT-rGO electrode. As shown in Table 1, as the degree of reduction of GO increases with increasing annealing temperature, the specific surface area of T-rGO decreases. This could be due to the formation of a structure similar to graphite as more oxygen groups are removed at higher annealing temperatures. By contrast, the specific surface area of BT-rGO increases with increasing annealing temperature. This means that the substitutional incorporation of boron atoms can provide defect-like small pores in the basal plane of the graphene sheet, inhibiting the formation of the graphitic structure. This is also consistent with the results of Raman analysis, which indicated that defects were derived from boron doping. Based on these results, it can be concluded that the high specific capacitance of BT-rGO is due to its enhanced electrical conductivity and high specific surface area derived from the boron doping.

In summary, we herein demonstrated that boron-doped graphene nanoplatelets (BT-rGO) can be prepared by the simultaneous reduction and doping of GO using simple thermal annealing with g-B_2_O_3_ as a dopant. At a high annealing temperature, the oxygen-containing groups of GO are removed, and defects thus obtained can act as active sites for the reaction between carbon and boron atoms released by thermal decomposition of g-B_2_O_3_. BT-rGO annealed at 1000 °C shows the maximum boron concentration of 6.04 ± 1.44 at %, which is the highest value among B-doped graphenes produced by CVD, arc discharge or the substitutional doping of GO or rGO. Boron atoms are uniformly doped into the graphene networks and the amount of boron can be easily controlled by changing the annealing temperature. Furthermore, this process is suitable for gram-scale production of B-doped graphene nanoplatelets using laboratory equipment. Therefore, B-doped graphene nanoplatelets could be utilized in various energy storage and conversion applications requiring three-dimensional nanostructures and facile synthesis. We also demonstrated, as a proof-of-concept, a highly-B-doped graphene electrode in an electrochemical double-layer capacitor (EDLC). B-doped graphene nanoplatelets have an improved electrical conductivity due to the increased charge carrier concentration derived from boron doping and defect-like small pores in the basal plane of the graphene sheet. This improved conductivity leads to a high specific capacitance of 448 F/g without the use of conductive additives such as carbon black. We believe that B-doped graphene nanoplatelets can be used in other applications such as biological/chemical sensors, electrocatalyst and nano-electronics because of their reliable and controllable electrical properties regardless of the outer environment, unlike N-doped graphene nanoplatelets.

## Methods

### Preparation of thermally reduced graphene oxide with boron doping (BT-rGO) or without boron doping (T-rGO)

Natural grade, ~100 mesh, 99.9% (metals basis) graphite powder was purchased from Asbury Graphite Mills, Inc. The 98% H_2_SO_4_ and reagent-grade B_2_O_3_ were purchased from Sigma-Aldrich, and KMnO_4_ and 30% H_2_O_2_ aqueous solution were obtained from Yakuri. All chemicals were used without further purification. Graphene oxide (GO) was prepared via the modified Hummers method using expandable graphite flake as a starting material^43^. The as-prepared GO suspension (40 mL) with a concentration of 0.5 mg/mL in water was well mixed with boron oxide (B_2_O_3,_ 20 mg) using an ultrasonicator for 1 h. Then the suspension was dried using a freeze dryer (Operon FDCF-12001) and subjected to thermal annealing at 300, 500, 700 or 1000 °C using a tubular furnace in an N_2_ atmosphere for 1 h. Finally, the obtained black powder was washed with hot distilled water and filtered, and then this procedure was repeated three times in order to remove the residual boron oxide. Thermally reduced GO without boron substitution (T-rGO) was prepared as a control. In brief, 40 mL of GO suspension was dried using a freeze dryer and subjected to thermal annealing as described for the preparation of BT-rGO. The obtained T-rGO black powder was also washed and filtered as described above.

### Characterization

The surface morphology and thickness of GO and BT-rGO were characterized using an atomic force microscope (AFM, MFP3D, Asylum Research) at room temperature in non-contact mode with 10 nm standard cantilevers (AC160TS, Olympus). The structural and chemical composition of BT-rGO were characterized using a transmission electron microscope (TEM, Titan 80-300^TM^, FEI) with an accelerating voltage of 300 kV and electron energy loss spectroscopy (EELS). X-ray photoelectron spectroscopy (XPS) measurements were carried out on a PHI 5000 VersaProbe (Ulvac-PHI) using monochromatic Al Kα radiation in order to quantitatively and qualitatively characterize BT-rGO and T-rGO. Raman spectroscopy (LabRam ARAMIS, Horiba Jobin-Yvon) was also performed for characterization. The specific surface areas of T-rGO and BT-rGO were determined using the Brunauer–Emmett–Teller method (BET, Belsorp max, BEL Japan) with liquid nitrogen. Electrical conductivity measurements were carried out using a four-probe system consisting of a four-point cylindrical probe head (JANDEL Engineering Ltd.), a direct current precision power source (Model 6220, Keithley), and a nanovoltmeter (Mode 2182A, Keithley). Cyclic voltammetry (CV, EDAQ e-corder 401) was performed to characterize the electrochemical properties with a three-electrode system with an aqueous 6 M KOH. A Pt plate and Hg/HgO served as a counter and reference electrodes, respectively. To fabricate a working electrode, BT-rGO powder (or T-rGO) and polyvinylidene fluoride (PVDF, Sigma-Aldrich) were mixed in a mass ratio of 95:5 and dispersed in *N*-methylpyrrolidone (NMP) solvent using a paste mixer. The resulting paste was spread onto nickel foam (1 cm × 1 cm) and dried at 120 °C under vacuum. The prepared electrode was immersed in DI water as a non-solvent for 12 h to remove NMP solvent where exchange occurred between NMP and water^42^. The CV tests were performed between −0.9 and −0.1 V vs. Hg/HgO at different scan rates of 10, 20, 50, 100, and 200 mV/s. The specific capacitance (C, F/g) of the working electrode was calculated according to C = (∫*IdV*)/(*vmV*), where *I* is the response current density (A/cm^2^), *V* is the potential, *v* is the potential scan rate (mV/s), and *m* is the mass of the electroactive materials in the electrodes (g). The mass (*m*) of the prepared electrode determined by a microbalance was approximately 1 mg/cm^2^.

## Author Contributions

D.-Y. Y. and W. J. synthesized materials, N. D. K. and S. Y. Y. measured the supercapacitive properties, S.-S. L. and B. J. S. analyzed the electrical properties, and H. C. characterized materials by TEM. J. A. L. and H. K. planned the work, evaluated the mechanism and wrote the manuscript.

## Additional Information

**How to cite this article**: Yeom, D.-Y. *et al.* High-concentration boron doping of graphene nanoplatelets by simple thermal annealing and their supercapacitive properties. *Sci. Rep.* **5**, 9817; doi: 10.1038/srep09817 (2015).

## Supplementary Material

Supporting Information Supplementary Figures 1-6

## Figures and Tables

**Table 1:** Electrical Conductivities and BET Data for T-rGO and BT-rGO Annealed at Various Temperatures.

**T-rGO**	**Electrical Conductivity(S/cm)**	**BET(m**^**2**^**/g)**	**BT-rGO**	**Electrical Conductivity(S/cm)**	**BET(m**^**2**^**/g)**
300	0.051	69.9	300	0.85	63.5
500	0.053	61.8	500	1.34	75.2
700	0.22	60.5	700	9.46	81.5
1000	1.81	50.4	1000	44.34	122.4
