# Supplementary material for: High-concentration boron doping of graphene nanoplatelets by simple thermal annealing and their supercapacitive properties
Source: Sci Rep. 2015 May 5;5:9817. doi: 10.1038/srep09817 (PMC4419459; doi:10.1038/srep09817)
Supplement: Supporting Information — Supplementary Figures 1-6 [file srep09817-s1.doc]

Supporting Information for

High-concentration boron doping of graphene nanoplatelets by simple thermal annealing and their supercapacitive properties

Da-YoungYeom^1,2^ , Woojin Jeon^1^ , Nguyen Dien Kha Tu^1,3^ , SoYoungYeo^4^, Sang-Soo Lee^1,5^, Bong June Sung^2^ , Hyejung Chang^6^, Jung Ah Lim^3,4^ & Heesuk Kim^1,3^

^1^Photo-electronic Hybrids Research Center, Korea Institute of Science and Technology (KIST), Seoul 136-791, Korea.

^2^Department of Chemistry, Sogang University, Seoul 121-742, Korea.

^3^Nano-Materials and Engineering, Korea University of Science and Technology (UST), Dae-Jeon, Korea.

^4^Interface Control Research Center, Korea Institute of Science and Technology (KIST), Seoul 136-791, Korea.

^5^KU-KIST Graduate School of Converging Science and Technology, Korea University, Seoul 136-701, Korea.

^6^Advanced Analysis Center, Korea Institute of Science and Technology (KIST), Seoul 136-791, Korea


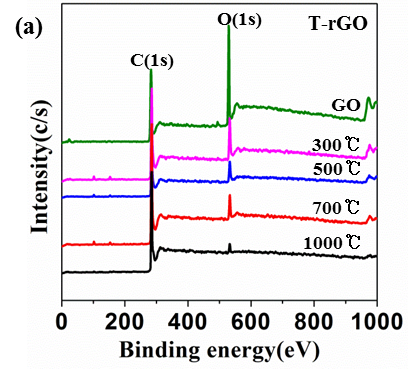

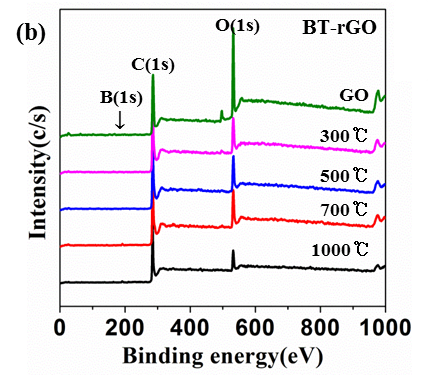


Figure S1. XPS survey spectra of (a) T-rGO and (b) BT-rGO.


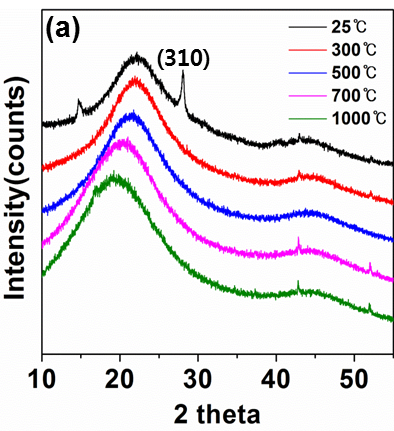

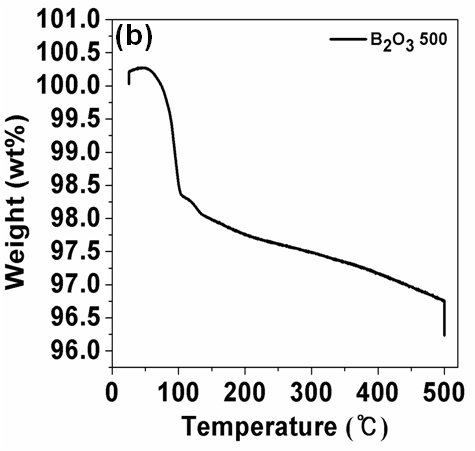


*
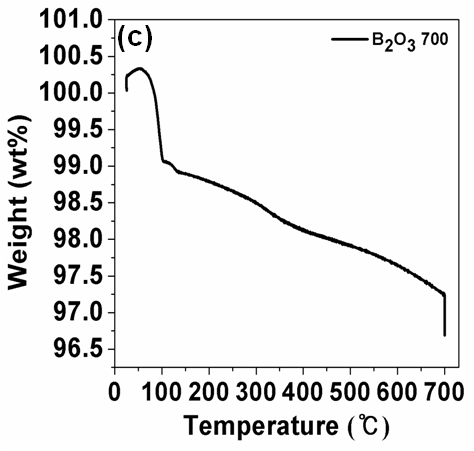

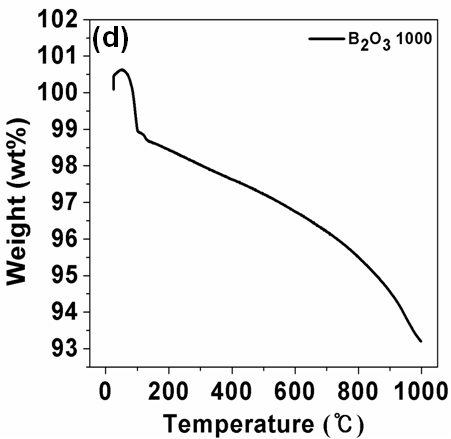
*

Figure S2. (a) XRD patterns of g-B_2_O_3_, and (b), (c) and (d) TGA data of g-B_2_O_3_ (The temperature increases up to the desired point (500, 700 and 1000 °C) and remains at that temperature for 1 h).


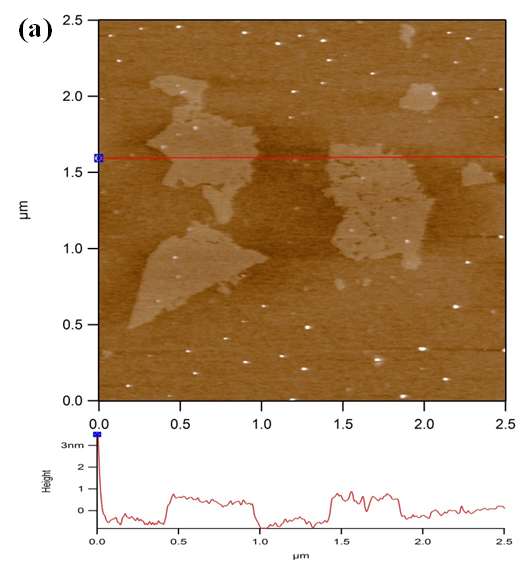

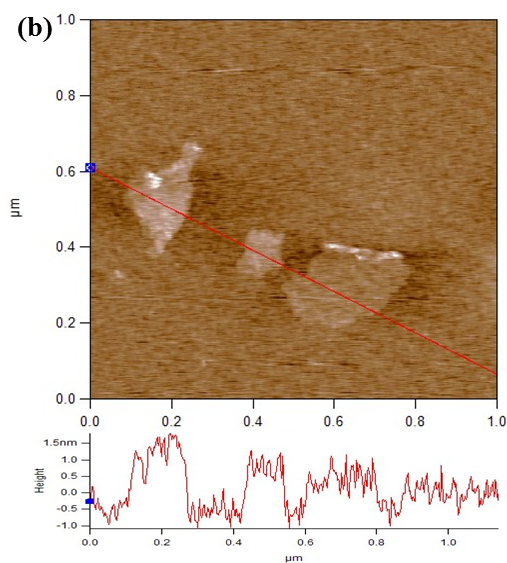


Figure S3. AFM images of (a) GO and (b) BT-rGO prepared at 1000 °C.


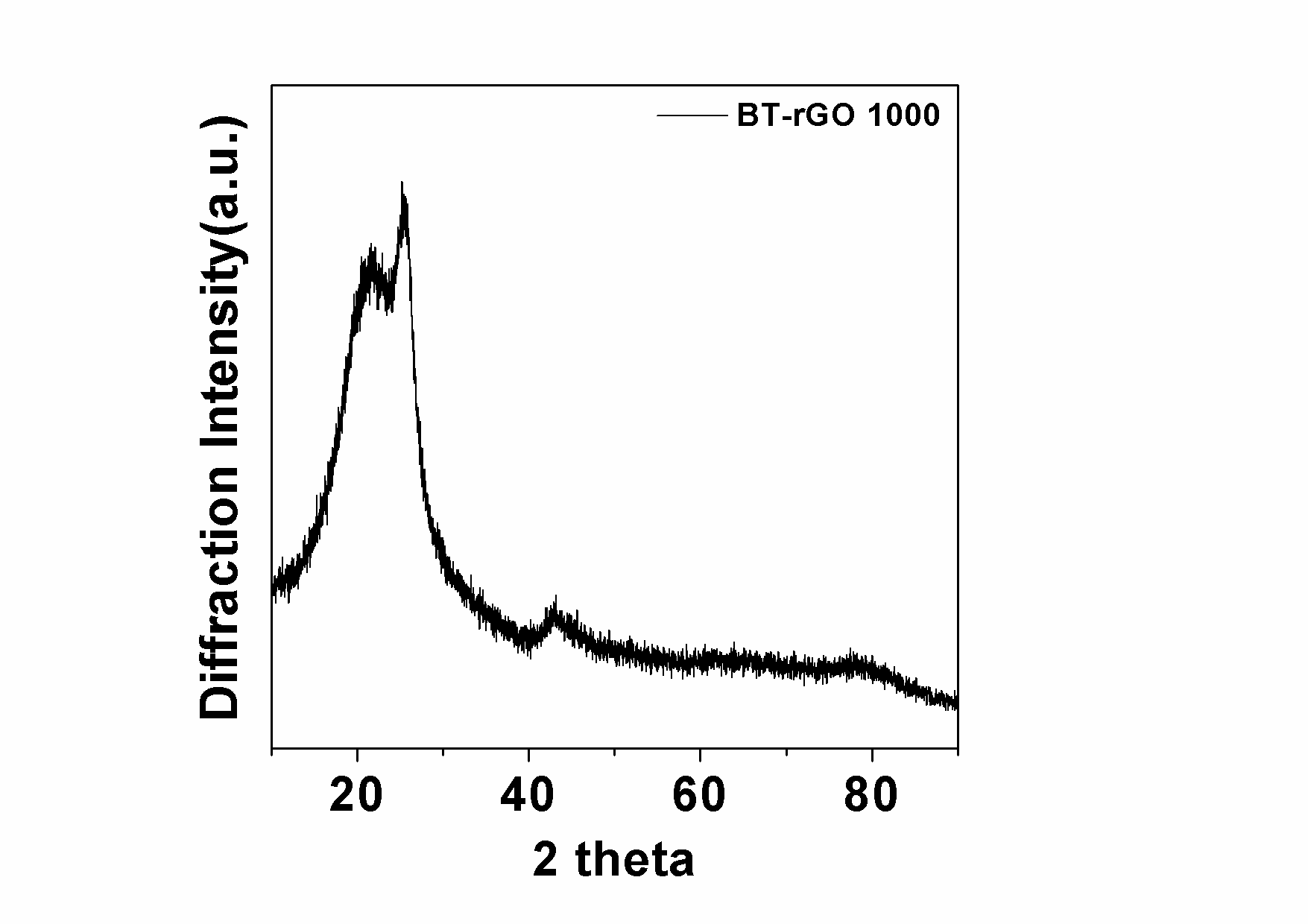


**(002)**

**(100)**

Figure S4. XRD pattern of BT-rGO prepared at 1000 °C.


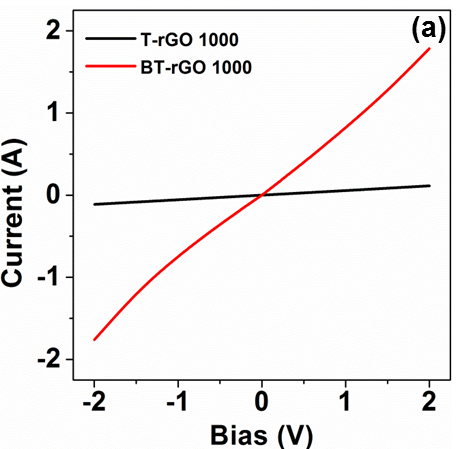

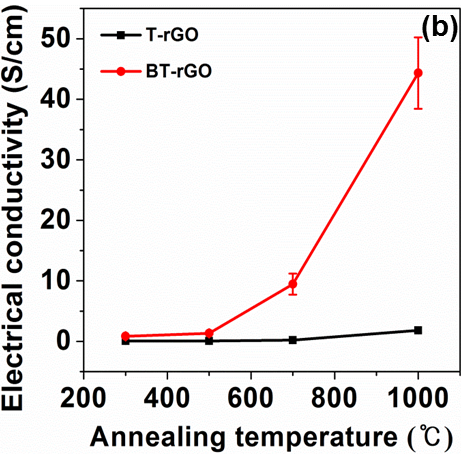


Figure S5. (a) Current-voltage (I-V) curves of T-rGO and BT-rGO prepared at 1000 °C, and (b) electrical conductivity of T-rGO and BT-rGO as a functional of annealing temperature.


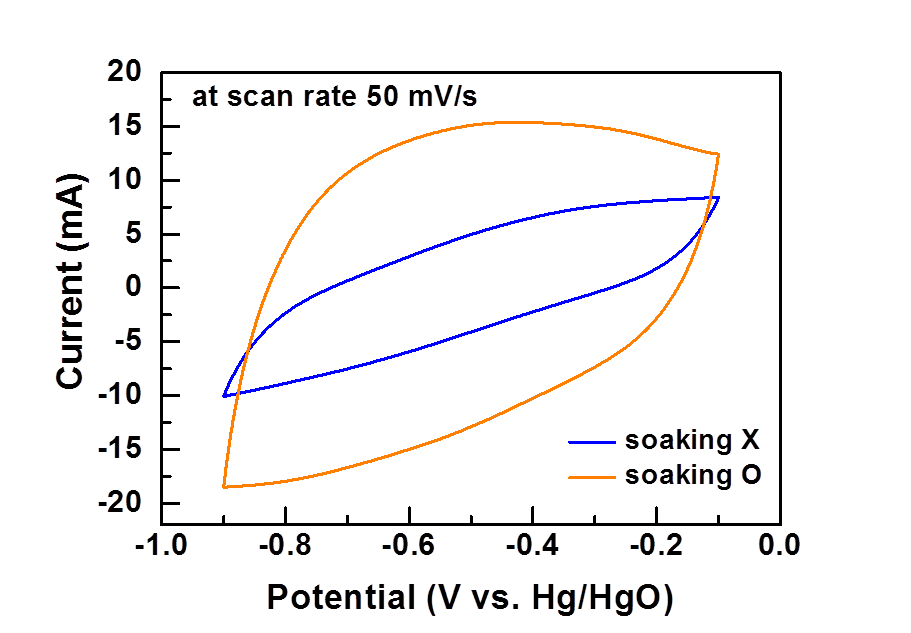


Figure S6. Cyclic voltammetry (CV) curves of BT-rGO (prepared at 1000 °C) with and without soaking in DI water.
